# Rural patient and provider perceptions of telehealth implemented during the COVID-19 pandemic

**DOI:** 10.1186/s12913-023-09994-4

**Published:** 2023-09-12

**Authors:** David Klee, Derek Pyne, Joshua Kroll, William James, Kelly A. Hirko

**Affiliations:** 1 Munson Medical Center, Munson Healthcare, Traverse City, MI USA; 2 Department of Family Medicine, College of Human Medicine, Michigan State University, East Lansing, MI USA; 3 1400 Medical Campus Drive, Traverse City, MI 49684 USA; 4 Department of Epidemiology and Biostatistics, College of Human Medicine, Michigan State University, East Lansing, MI USA

**Keywords:** Telehealth, Telemedicine, COVID-19, Rural, Implementation, Provider, Barriers

## Abstract

**Background:**

Understanding perceptions of telehealth  implementation from patients and providers can improve the utility and sustainability of these programs, particularly in under-resourced rural settings. The purpose of this study was to evaluate both patient and provider perceptions of telehealth visits in a large rural healthcare system during the COVID-19 pandemic. To promote sustainability of telehealth approaches, we also assessed whether the percentage of missed appointments differed between in-person and telehealth visits.

**Methods:**

Using anonymous surveys, we evaluated patient preferences and satisfaction with telehealth visits from November 2020 -March 2021 and assessed perceptions of telehealth efficiency and value among rural providers from September–October 2020. We examined whether telehealth perceptions differed according to patients’ age, educational attainment, insurance status, and distance to clinical site and providers’ age and length of time practicing medicine using ANOVA test. We also examined whether the percentage of missed appointments differed between in-person and telehealth visits at a family practice clinic within the rural healthcare system from April to September 2020 using a Chi-square test.

**Results:**

Over 73% of rural patients had favorable perceptions of telehealth visits, and satisfaction was generally higher among younger patients. Patients reported difficulty with scheduling follow-up appointments, lack of personal contact and technology challenges as common barriers. Over 80% of the 219 providers responding to the survey reported that telehealth added value to their practice, while 36.6% agreed that telehealth visits are more efficient than in-person visits. Perception of telehealth value and efficiency did not differ by provider age (*p* = 0.67 and *p* = 0.67, respectively) or time in practice (*p* = 0.53 and *p* = 0.44, respectively). Technology challenges for the patient (91.3%) and provider (45.1%) were commonly reported. The percentage of missed appointments was slightly higher for telehealth visits compared to in-person visits, but the difference was not statistically significant (8.7% vs. 8.0%; *p* = 0.39).

**Conclusions:**

Telehealth perceptions were generally favorable among rural patients and providers, although satisfaction was lower among older patients and providers. Our findings suggest that telehealth approaches may add value and efficiency to rural clinical practice. However, technology issues for both patients and providers and gaps in care coordination need to be addressed to promote sustainability of telehealth approaches in rural practice.

**Supplementary Information:**

The online version contains supplementary material available at 10.1186/s12913-023-09994-4.

## Introduction

Telehealth approaches hold tremendous potential to address healthcare access barriers, particularly in geographically disperse rural settings, where patients often travel longer distance to receive care [1–3]. The efficacy of telehealth for provision of clinical care has been demonstrated in multiple settings [4–8]. In a systematic review of the literature, telehealth visits were consistently associated with high patient satisfaction and improved clinical outcomes [9]. Moreover, telehealth visits have been shown to add quality and value to clinical practice by improving efficiency, enhancing convenience for patients and providers alike, [10] and reducing provider stress and burnout [11, 12]. Burgeoning evidence also suggests that telehealth approaches may enable accessible care by reducing missed healthcare appointments [13].

Despite the well-documented benefits of telehealth approaches, numerous barriers have impeded the widespread adoption of telehealth in clinical care. Commonly reported barriers to telehealth include the necessity of physical exams, technological literacy, cost and reimbursement challenges, and privacy and security concerns [14–16]. Nevertheless, the use of telehealth visits in clinical practice has increased in recent years, doubling from 14% of visits in the US in 2016 to 28% in 2019, [10] and expanding rapidly in response to the COVID-19 pandemic [17–19]. The rapid adoption of telehealth in clinical practice has been bolstered by expanded reimbursements for telehealth visits in an effort to promote continued access to care and prevent COVID-19 transmission [19, 20].

Important lessons from this period of deregulation can be used to inform post-pandemic telehealth regulations and maintain the momentum of telehealth approaches moving forward [19]. Indeed, given the substantial investment in telehealth infrastructure and training in response to the COVID-19 pandemic, there is strong impetus for health systems to systematically evaluate telehealth implementation efforts and to characterize perceptions of telehealth from both patient and provider perspectives. This is particularly relevant for rural populations who arguably stand to benefit most from telehealth approaches [1–3]. However, to our knowledge, no prior studies have simultaneously examined rural provider and patient perspectives of telehealth implemented during the COVID-19 pandemic. It is also unclear whether the transition to telemedicine has impacted the likelihood of missed healthcare appointments in rural clinical settings.

Thus, the purpose of this study was to evaluate patient and provider perceptions of telehealth visits implemented at a large rural healthcare system during the COVID-19 pandemic. We sought to identify patient and provider barriers that may limit telehealth’s broader adoption, and assessed whether the percentage of missed appointments differed between in-person and telehealth visits.

## Materials and methods

### Study setting and procedures

This study was conducted at Munson Healthcare (MHC), a large rural healthcare system serving over 500,000 residents across 30 rural counties in Northwest Michigan. The patient survey was distributed at Munson Family Practice Center (MFPC), a MHC-owned family practice office located directly adjacent to Munson Medical Center, the flagship community hospital of the MHC system.

All patients seen during the study period 11/2/2020 to 3/24/2021 were asked by office staff at telehealth visit check-in to complete the voluntary and anonymous online survey to assess telemedicine perceptions. Those who agreed to participate were sent a secure link to the survey following completion of their visit. With a MFPC patient population of 12,000, we aimed to collect 100 surveys to promote representativeness of the target patient population, using a confidence level of 95% and a 10% margin of error. Providers across the MHC system were sent an emailed link from the MHC digital health team to complete the voluntary and anonymous online provider survey. The online survey was developed using SurveyMonkey and the survey link was distributed via email from the MHC telehealth coordinator to 1,180 MHC providers and MHC-affiliated and independent healthcare providers from September 1, 2020 to October 1, 2020. A retrospective review of electronic health records for all patients with scheduled appointments at Munson Family Practice Center from April 2020 to September 2020 was conducted to assess the number missed visits by visit type (telehealth vs. in-person) as noted in the medical record.

### Patient survey

The 12-question survey was based on a survey used in a prior study, [21] and assessed telehealth use, reason for use, and telehealth perceptions and barriers. Participants were asked to rate perceptions of telehealth on a Likert-scale ranging from “Strongly Disagree” to “Strongly Agree”. Perceptions of telehealth were assessed using the following statements; “I like using telehealth visits”, “Telehealth visits are convenient”, “I am likely to request a telehealth visit in the future”, “I received high quality care”, “There was an efficient process for check-in”, “There was an efficient process for check-out/follow up”, “It is important that my healthcare provider be physically in the room”, “I felt comfortable with the plan of care and follow up”, “Telehealth was a reasonable way to maintain social distancing and stay at home orders during the COVID-19 pandemic while receiving health care services”, I am glad that I had the option for telehealth offered to me during the COVID-19 pandemic.” Participants were also asked to select reasons for liking telehealth from the following options: “I feel safer by reducing person-to-person contact during COVID-19″, “Less travel time”, “More convenient for my personal schedule”, “I do not need additional child care services”, “Less waiting time between check-in and seeing provider”. Participants were also asked to select reasons for disliking telehealth visits from the following options: “I did not have personal contact with my healthcare provider”, “I had technology problems or interruptions”, “Follow up was difficult to schedule”, “There was a barrier to communication (example: difficulty in accessing Medical Translation Services)”, “Other (please specify)”. Participants were asked to specify reasons for requesting telehealth visits in the future, and reasons why they would not request a telehealth visit in the future using open-ended text responses. Finally, participants were asked whether they experienced any of the following technical problems or barriers with telehealth visits; “Lack of smart phone, tablet, or computer”, “Internet/WIFI difficulties”, “Software or app problems”, “I have not experienced any technical problems/barriers”. Information on demographic factors (age, educational attainment, health insurance status, and approximate travel time in minutes from home to the clinic) were also ascertained.

### Provider survey

We utilized a 14-question survey generated from a prior study conducted by the American Medical Association [10] to assess telehealth preferences and impact on clinical practice. Inclusion criteria included all providers including physicians, nurse practitioners, and physician assistants who had participated in either a video or telephone visit at MHC in the past 3 months. The primary outcome of interest was the perception of quality and value that telehealth visits may have added to rural clinical practice. Secondary outcomes evaluated included barriers to use and provider’s perceptions on the types of visits where telehealth worked well (e.g., acute care, Medicare wellness).

### Statistical analyses

Descriptive statistics were used to summarize the patient and provider survey data. We used ANOVA tests to evaluate differences in perception of telehealth visits according to patient age, educational attainment, insurance status, distance to the clinic, and reason for clinic visit. We assessed whether provider telehealth perceptions significantly differed by provider age and length of time practicing medicine using ANOVA tests with *p*-values < 0.05 considered significant. In secondary exploratory analyses, we compared the percentage of all missed visits across groups defined by visit type (telehealth vs. in-person) using Chi-square tests with *p*-values < 0.05 considered significant. All analyses were performed with SAS version 9.4 statistical software. The study was approved by the Munson Healthcare Institutional Review Board.

## Results

### Patient characteristics

Overall, 100 patients responded to the patient survey, with 93 providing information on telehealth perceptions included in this study analysis. As shown in Table 1, 56% of patients surveyed were between 35 and 64 years of age and nearly a quarter of the rural patient population travelled over 30 min to the clinic. Over half of patient participants had high school education or less, and 25% received Medicaid. Study participants were seen virtually for a variety of reasons including for medication review (22.6%), to report a new problem (20.4%), follow-up on health issue (17.2%), new patient visit (17.2%), transition of care (14.0%), behavioral health (12.9%) and other wellness and/or rehabilitation check in (3.3%).

**Table 1:** Characteristics of rural patients (*n* = 93) and providers (*n* = 219)

	**Patients** **N (%)**		**Providers** **N (%)**
**Age, years**		**Age, years**^**c**^	
18–25	6 (6.5%)	25–30	13 (6.9%)
26–34	21 (22.2%)	31–40	44 (23.3%)
35–50	22 (23.7%)	41–50	57 (30.2%)
51–64	30 (32.3%)	51–65	67 (35.5%)
> 65	14 (15.1%)	> 65	8 (4.2%)
**Educational Attainment**		**Years Practicing Medicine**^**c**^	
< High School	3 (3.2%)	Resident	14 (7.4%)
High School Graduate	48 (51.6%)	< 5	26 (13.8%)
Some College	22 (23.7%)	5–10	24 (12.7%)
Bachelor’s Degree	17 (18.3%)	11–20	50 (26.5%)
Graduate School	3 (3.2%	21–30	55 (29.1%)
		> 30	20 (10.6%)
**Insurance Status**^**a**^			
No Insurance	6 (3.2%)	**Type of Practice**^**d**^	
Medicare	14 (15.2%)	Munson Healthcare owned	104 (54.7%)
Medicaid	23 (25.0%)	Independent	86 (45.3%)
Commercial	49 (53.3%)		
**Time Spent Travelling to Clinic**			
10 min or less	3 (3.2%)		
11–20 min	24 (25.8%)		
21–30 min	44 (47.3%)		
More than 30 min	22 (23.7%)		
**Reason for Visit**^**b**^			
Transition of care	13 (14.0%)		
Medication review	21 (22.6%)		
Behavioral health	12 (12.9%)		
New patient visit	16 (17.2%)		
Report new problem	19 (20.4%)		
Follow-up on health issue	16 (17.2%)		
Wellness/rehabilitation check	3 (3.3%)		

^a^Missing information on insurance status for 1 patient

^b^Number of patients exceeds the sample size as patients could list multiple reasons

^c^Missing information on age and years practicing medicine for 30 providers

^d^Missing information on type of practice for 19 providers

### Provider characteristics

For the provider survey, 252 providers (24.8%) completed the survey, with 219 providers (86.9%) reporting telehealth use in the past 3 months included in this analysis. The age range of providers was normally distributed, with nearly 66% of providers between the ages of 41–65 years (Table 1). The majority of providers had been practicing medicine for 11–20 years (26.5%) or between 21–30 years (29.1%) and most were MHC providers (54.7%).

### Patient perceptions

Patient participants had favorable perceptions of telehealth visits, with over 73% either agreed or strongly agreed that they liked using telehealth (Table 2). 97 percent of patients strongly agreed/agreed telehealth is convenient and 94% strongly agreed/agreed that telehealth offers a reasonable option to maintain social distancing during the pandemic. Patients’ responses were less favorable with regard to the efficiency of the check-out process with only 28% strongly agreed/agreed that the process was efficient, yet 47% of patients strongly agreed/agreed they were likely to request a telehealth visit in the future. Nearly 50% of patients agreed or strongly agreed that it was important to have their healthcare provider be physically in the room. Interestingly, favorable overall perceptions of telehealth visits differed according to patient age, with generally higher favorability among younger patients. For example, patients ages 18–25 years reported average score of 4.5 on the statement “I like using telehealth visits” (on scale of 1 = strongly disagree to 5 = strongly agree), compared to 3.5 among those 51–64 years and 4.0 among those 65 years and older; *p* = 0.007). Overall telehealth perception did not vary significantly according to patient’s educational attainment (*p* = 0.15), insurance status (*p* = 0.72), distance travelled to the clinic (*p* = 0.49), or according to the type of telehealth visit (*p* = 0.10).

**Table 2:** Rural patient perceptions of telehealth visits (*n* = 93)

	**Strongly Agree**	**Agree**	**Neutral**	**Disagree**	**Strongly Disagree**
I like using telehealth visits	33.3%	39.8%	20.4%	4.3%	2.2%
Telehealth visits are convenient	44.1%	52.7%	3.2%	0.0%	0.0%
I am likely to request a telehealth visit in the future	18.3%	29.0%	38.7%	9.7%	4.3%
I received high quality care	12.9%	51.6%	32.3%	2.2%	1.1%
There was an efficient process for check-in	9.7%	54.8%	28.0%	7.5%	0.0%
There was an efficient process for check-out	4.3%	14.0%	38.7%	40.9%	2.2%
It is important that my healthcare provider be physically in the room	7.5%	39.8%	50.5%	2.2%	0.0%
I felt comfortable with the plan of care and follow-up	6.5%	63.4%	25.8%	4.3%	0.0%
Telehealth was a reasonable way to maintain social distancing and stay at home orders during the COVID-19 pandemic while receiving healthcare services	49.5%	44.1%	6.5%	0.0%	0.0%
I am glad that I had the option for telehealth offered to me during the COVID-19 pandemic	40.9%	33.3%	18.3%	5.4%	2.2%

### Provider perceptions

Providers’ perceptions of telehealth visits are shown in Table 3. Over 80% of providers strongly agreed or agreed that telehealth added value to their practice. The perception of telehealth efficiency was slightly lower than perceived value, with 36.6% of providers strongly agreeing (12.9%) or agreeing (23.7%) that a telehealth visit is more efficient than an in-person visit. Perception of telehealth value and efficiency did not differ significantly by providers’ age (*p* = 0.67 and *p* = 0.67, respectively) or time in practice (*p* = 0.53 and *p* = 0.44, respectively). However, the perception of telehealth’s value was generally lower among older providers, with 84.6% of providers aged 25–30 years and only 57.2% of providers aged 65 years and older agreeing or strongly agreeing that telehealth visits added value to practice. The majority of providers strongly agreed or agreed that telehealth impacted clinical practice by improving access to care (90.1%), timeliness of care (70.4%), the safety of patients (66.4%) and patient and family-centered care (54.4%). The types of medical visits most highly suited for telehealth in this study were anxiety/depression, mental health visits, COVID-19 assessments, and Diabetes. Providers in this study predicted continued use of telehealth, with just over half selecting the range of 1–10% for the amount of their patient visits which would be conducted virtually moving forward.

**Table 3:** Rural provider perceptions of telehealth visits (*n* = 219)

	**Strongly Agree**	**Agree**	**Neutral**	**Disagree**	**Strongly Disagree**
Adds Value	33.2%	47.2%	10.9%	7.3%	1.6%
Adds Efficiency	12.9%	23.7%	25.8%	27.8%	9.8%
**Telehealth has impacted my practice by improving:**					
The health of my patients	11.7%	35.6%	39.9%	12.8%	0.0%
The safety of my patients	19.2%	47.2%	21.8%	7.8%	4.2%
Timeliness of care of my patients	16.7%	53.7%	21.4%	5.2%	3.1%
Patient and Family Centered-Care	12.3%	42.1%	29.2%	12.9%	3.5%
Access to care	30.9%	59.2%	4.2%	3.7%	2.1%
No-show rate	11.4%	22.3%	45.7%	17.7%	2.9%
Financial health of my practice	6.6%	25.6%	43.5%	14.9%	9.5%
Job satisfaction	10.5%	22.5%	38.7%	19.4%	8.9%
**Types of visits where telehealth works well:**					
Diabetes	19.8%	53.2%	15.1%	11.9%	0.0%
Anxiety/Depression	44.7%	42.1%	8.6%	3.3%	1.3%
Other Mental Health	36.7%	48.3%	10.9%	2.0%	2.0%
Hypertension	9.6%	30.2%	29.4%	26.5%	4.4%
Coronary Artery Disease	13.0%	36.6%	27.5%	22.9%	0.0%
Asthma/COPD	7.3%	22.6%	27.0%	38.7%	4.4%
Medicare Wellness	33.3%	30.1%	11.4%	19.5%	5.7%
Skin Lesion/Rash	6.5%	30.7%	21.6%	28.8%	12.4%
Acute Care	9.6%	37.0%	19.9%	27.4%	6.2%
Hospital/ED follow-up	5.7%	35.9%	21.4%	20.1%	5.7%
COVID-19 Assessment	35.2%	38.6%	17.2%	7.6%	1.4%

### Reported challenges with telehealth visits

Challenges were encountered with telehealth visits among both patients and providers in this study (Table 4). Patients commonly cited difficulty with scheduling follow-up (48.3%) and lack of personal contact (46.0%), with only 12.6% reporting technology problems (software issues, lacking smart phone, tablet, computer, and/or broadband internet access) and 2.3% reporting communication barriers. However, 91.3% of providers reported technology challenges for the patient, and 45.1% reported technology challenges for the provider. Moreover, providers also cited barriers related to lack of reimbursement (34.4%) and lack of implementation support (20.0%).

**Table 4:** Rural patient and provider reported challenges with telehealth visits

	**Patients** **N (%)**
Did not have personal contact with provider	40 (46.0%)
Technology problems or interruptions	11 (12.6%)
Follow-up was difficult to schedule	42 (48.3%)
Communication barrier	2 (2.3%)
Other	24 (27.6.0%)
None reported	9 (10.3%)
	**Providers** **N (%)**
Lack of reimbursement	67 (34.4%)
Licensure	4 (2.1%)
Technology challenges for the patient	178 (91.3%)
Technology challenges for the provider/practice	88 (45.1%)
Low patient engagement	37 (19.0%)
Lack of implementation support	39 (20.0%)
No challenges	2 (1.0%)

Percentages do not add to 100% as respondents could choose multiple options

### Missed appointments

**Fig. 1:**
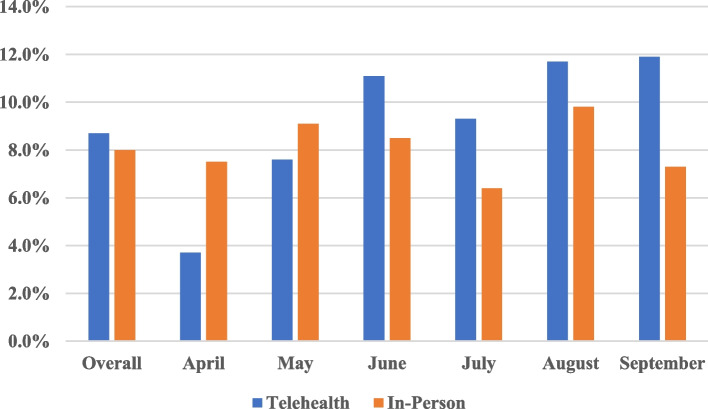
Percentage of Missed Appointments Overall and by Month According to Visit Type (Telehealth vs. In-Person)

Overall, 6,604 visits were scheduled at Munson Family Practice from April through September 2020. The majority of clinical visits were scheduled in-person (*n* = 3,832, 58.0%), while 42.0% (*n* = 2,772), of visits were scheduled via telehealth. A total of 547 visits were missed over the study period (240 telehealth and 307 in-person). As shown in Fig. 1, the percentage of missed appointments out of all scheduled visits was slightly higher for telehealth visits compared to in-person visits, but the difference was not statistically significant (8.7% vs. 8.0%; *p* = 0.39). For telehealth visits, the percentage of no-shows generally increased from 3.7% in April to 11.9% in September. The percentage of no-shows for in-person visits also increased slightly across study months, ranging from 6.4% in July to 9.8% in August. Among patients with missed appointments, we did not observe any differences in patient age according to visit type (*p* = 0.35).

## Discussion

In this study, rural patients had generally favorable perceptions of telehealth, and satisfaction was higher among younger patients. Rural providers overwhelmingly reported that telehealth added value to clinical practice, though less than half of providers felt that telehealth improved efficiency. Difficulties with scheduling follow-up appointments, lack of personal contact and technology challenges were commonly reported barriers to telehealth. We did not observe any differences in the percentage of missed appointments according to visit type (telehealth vs. in-person) in this study. Taken together, our findings suggest that telehealth approaches are acceptable to rural patients and providers but that technology issues and gaps in care coordination need to be addressed to promote sustainability.

Our finding that favorability of telehealth was higher among younger compared to older patients were consistent with results from prior studies conducted both before [22], and during the COVID-19 pandemic [23] and may be attributed in part to lower technology access and lower digital literacy among older adults [24]. While results from a prior study conducted in 2019 demonstrated lower satisfaction with telehealth among those with less educational attainment, [23] in this study of rural patients, perceptions of telehealth during the pandemic did not vary according to educational attainment, insurance status, distance travelled to clinic or according to type of visit. The benefits of telehealth approaches for older adults have been well documented and include increased convenience, care partner engagement, and improved understanding of home environments by clinicians [22]. Therefore, additional resources are needed to support older adults and those with limited access to internet and technology to maintain quality care in telehealth settings and to avoid exacerbating existing health disparities among older and underserved populations [18].

The provision of quality clinical care relies on a trusted exchange of information between the patient and provider, traditionally occurring through face-to-face clinic visits. With the rapid adoption of telehealth approaches in recent years, it is still unclear how virtual approaches impact communication and the overall patient-provider relationship. One study reported that the majority of providers felt that the physician–patient relationship was unimpaired using telehealth approaches implemented during the pandemic [24]. However, another recent study of telehealth during the pandemic, which used semi-structured interviews found that the physicians noted concerns about loss of personal connections with patients, difficulty reading people’s body language over videos and loss of connectiveness though physical touch [25]. Understanding patient perspectives is important to ensure that telehealth approaches are not detrimental to the relationship. In our study, nearly 40% of patients who reported disliking telehealth cited the lack of personal contact with their provider as the main reason. These findings suggest the need for future studies to address issues related to developing and maintaining the important physician–patient relationship in a virtual environment.

Most rural providers in this study believed that telehealth added value to clinical practice by improving the health and safety of patients, timeliness and access to care and patient and family-centered care—important goals for any outpatient clinical practice. These results were in accordance with findings from several recent studies conducted during the pandemic [24–28]. While we did not observe significant differences by provider age, the perception of telehealth’s added value generally decreased with increasing provider age in our study, with highest favorability among providers aged 25–30 years. As such, telehealth implementation efforts, particularly for an aging rural physician workforce,[29] may need to better understand and address age-related differences in telehealth perception. Importantly, telehealth implementation has been linked to less provider time spent in the electronic health records (EHR) outside of normal working hours [26]. Given that provider burnout has been correlated with time spent in the EHR outside of work, [30–32] this finding suggests that telehealth approaches could also help improve physician satisfaction and reduce burnout.

In this study, less than half of rural providers felt that telehealth approaches improved efficiency in clinical practice. These findings are discordant with results from several prior studies [24–27]. For example, the majority of providers in a large health system in central Pennsylvania reported that telehealth improved efficiency and cut down on driving time required to travel to other clinics [25]. Moreover, in an EHR-based study of over 600 providers in New York City, Beiser et al. observed increased efficiency in terms of the number of patients seen after implementation of telehealth [26]. Discrepancies in findings related to perceived efficiency could be due to the high level of technological challenges reported by rural providers in this study [24–27] Moreover, our study evaluated the rapid deployment of telehealth during the COVID-19 pandemic, and 20% of providers cited barriers related the lack of implementation support. These factors could also have contributed to the lower perception of telehealth efficiency observed in this study.

Reducing patient and provider barriers to telehealth will improve the overall quality of care with telehealth visits and promote broader telehealth adoption. Commonly reported barriers to telehealth identified in our study included difficulties with scheduling follow-up appointments, lack of personal contact and technology challenges. Telehealth approaches are highly dependent on broadband or cellular internet access, which is not equitably available [33–35]. For example, nearly 20% of the US population resides in rural communities, where access to academic medical centers, reliable internet and other resources is often limited [36, 37]. Given these challenges in rural settings, additional support may be necessary to limit technology challenges for both rural providers and patients. Interestingly, rural patients and providers reported divergent views of technology-related barriers in this study. Specifically, providers were more likely to report technology challenges for both patients and providers, while patient-reported technology challenges were less common. Additional studies are needed to explore potential underlying reasons for this discrepancy.

Reducing missed healthcare appointments is a key component to increasing efficiency in clinical practice [38]. In a prior qualitative study, providers cited more missed appointments in the virtual compared to in-person setting [22]. Missed healthcare appointments have been shown to be associated with multiple factors, including a lack of urgency to receive care, scheduling policy, fear and anxiety surrounding appointments, language barriers, forgetfulness, transportation-related issues, concern over service cost, weather, insurance coverage, long lead times to appointments, and miscommunication with clinic staff [39–41]. Results from our study suggested that telehealth visits did not significantly impact missed healthcare appointments in rural primary care settings during the pandemic. The lack of difference in missed appointments between telehealth and in-person visits in our study suggested that factors other than transportation-related issues may be more strongly associated with missed appointments in our rural setting. Although, preferences for telehealth may vary by geographic region – those living in metropolitan areas were less likely to miss telemedicine appointments but more likely to miss in-person appointments in a prior study, [13] potentially due to higher preference for accessing healthcare through technology and more robust internet access in urban areas. Future studies are needed to assess missed appointments according to visit type after the pandemic-associated social distancing measures were lifted.

One of the main strengths of this study is the focus on perceptions of rural patients and rural physicians across diverse provider settings, whereas most studies of telehealth during the pandemic have focused on urban regions and/or specific clinical subspecialties. Given that access to care can be particularly challenging in rural areas, rural populations arguably stand to benefit most from telehealth approaches. Thus, our study can provide insight to ensure the sustainability of telehealth approaches in rural regions, even after the pandemic. Additionally, findings from this study can be used to implement tailored telehealth approaches in rural health systems to improve quality of care and access. This study had several limitations, including potential survey response bias, given that only providers delivering telehealth and patients using telehealth were eligible for the study. We also lacked data on the volume of telehealth visits conducted by reason for visit, which could have influenced overall provider perceptions***.*** While the patient survey in this study was based on a similar survey from a prior publication, [21] the questions were not specifically validated. We were unable to determine the response rate for the patient survey due to pandemic-related limitations on tracking, although our sample size was calculated to promote broader representation of the target population. Lastly, generalizability of the results to other rural locations may be limited given that this survey study was conducted through a single rural healthcare system in Northwest Michigan.

## Conclusion

An improved understanding of patient and provider perceptions of telehealth is critical to widespread telehealth adoption and improved healthcare access. Findings from this study indicate favorable perceptions of telehealth among rural patients and providers and suggest that telehealth approaches may add value to rural clinical practice. However, technology issues for both patients and providers and gaps in care coordination need to be addressed to promote sustainability of telehealth approaches in rural practice. Given that most prior research evaluating telehealth implementation has been conducted in large urban healthcare settings, findings from this study are important and can be used to tailor telehealth approaches in other under-resourced settings. Further research is needed to more comprehensively characterize the underlying barriers and facilitators for telehealth and promote equitable implementation of telehealth programs in rural settings.

### Supplementary Information

**Additional file 1.** 

**Additional file 2.** 

## Data Availability

The datasets used and/or analyzed during the current study are available from the corresponding author on reasonable request.

## Abbreviations

ANOVA: Analysis of Variance
EHR: Electronic Health Records
MFPC: Munson Family Practice Center
MHC: Munson HealthCare
P: P-value
SAS: Statistical Analysis Software
US: United States
WIFI: Wireless Fidelity
