# Supplementary material for: Rural patient and provider perceptions of telehealth implemented during the COVID-19 pandemic
Source: BMC Health Serv Res. 2023 Sep 12;23:981. doi: 10.1186/s12913-023-09994-4 (PMC10496200; doi:10.1186/s12913-023-09994-4)
Supplement: Supplementary file 1 — Additional file 1. [file 12913_2023_9994_MOESM1_ESM.pdf]

Questionnaire

1. Patient demographics
   a) Age in years

      18-25

      26-34

      35-50

      50-65

      65 or older

   b) Education

      Did not graduate high school

      High School Diploma

      Some College to Associate’s Degree

      Bachelor’s Degree

      Graduate School or Higher

   c) Insurance

      No Insurance / Cash Pay

      Medicare

      Medicaid

      Commercial Insurance

   d) Approximate travel time, by private car, in minutes from home to MFPC

      10 minutes or less

      11-20 minutes

      21-30 minutes

      30 minutes or more

2. Reason for visit (select)

    TCM – Hospital Discharge Follow Up

    Medication Review

    Behavioral health

    Medicare Wellness

    New Patient

    Established patient: New problem

    Established patient: Follow up of existing problem

3. Scale of Like- Dislike of Televisit (1-5, strongly dislike to strongly like)

    1-2-3-4-5

4. Scale of Convenience 1 (least) -5 (most)

    1-2-3-4-5

5. Likelihood to request a video visit again 1 (least) -5 (most)

    1-2-3-4-5

6. Reasons for liking video visit (select all)

    I feel safer by reducing person-to-person contact during COVID-19

    Less Travel Time

    More convenient for my personal schedule

    I do not need additional child care services

    Less waiting time between check-in and seeing provider

    Other: ________

7. Reasons for disliking video visit

I did not have personal contact with my healthcare provider

I had technology problems or interruptions

Follow up was difficult to schedule

There was a barrier to communication, such difficulty in Medical Translation Services

Other: _____________

8. Reasons to request a video again (Free Response)

9. Reasons to not request video again (Free Response)

10. Technical problems or barriers – Select all that apply
    a) Lack of smart phone, tablet or computer
    b) Internet/Wifi difficulties
    c) Software (care-convene) problems
    d) Misc _____ Fill in blank

Agreement with statements

1 strongly disagree) 2 (disagree) 3 (neutral) 4 (agree) 5 (strongly agree)

1. I believe that I received high quality care

   1-2-3-4-5

2. I believe that there was an efficient process for check in

   1-2-3-4-5

3. I believe that there was an efficient process for check out/follow up

   1-2-3-4-5

4. I believe that it is important to have my healthcare provider be physically present in the room

   1-2-3-4-5

5. I felt comfortable with the plan of care and follow up

   1-2-3-4-5

6. I agree that telemedicine was a reasonable way to maintain social distancing and stay at home orders during the COVID-19 pandemic while receiving health care services

   1-2-3-4-5

7. I am glad that I had the option for telemedicine offered to me during the COVID-19 Pandemic

   1-2-3-4-5
