# Supplementary material for: Rural patient and provider perceptions of telehealth implemented during the COVID-19 pandemic
Source: BMC Health Serv Res. 2023 Sep 12;23:981. doi: 10.1186/s12913-023-09994-4 (PMC10496200; doi:10.1186/s12913-023-09994-4)
Supplement: Supplementary file 2 — Additional file 2. [file 12913_2023_9994_MOESM2_ESM.pdf]

MUNSON HEALTHCARE

Telehealth Research Project

Research Question: Do virtual visits add value and improve quality of care?

1. Have you used a telehealth platform (e.g., Care Convene, Cerner Community Works, Zoom for Healthcare, Doximity, Doxy.me, etc.) to conduct virtual patient care in the past 3 months?

- ◯ Yes
- ◯ No

MUNSON HEALTHCARE

Telehealth Research Project

2. For each of the visit types below, select on a Likert Scale which best applies: Telehealth works well for a __________ type of visit.

	Strongly Disagree	Disagree	Neutral	Agree	Strongly Agree	N/A
Diabetes	○	○	○	○	○	○
Anxiety/Depression	○	○	○	○	○	○
Other Mental Health	○	○	○	○	○	○
Hypertension	○	○	○	○	○	○
CAD	○	○	○	○	○	○
Asthma/COPD	○	○	○	○	○	○
Medicare Wellness	○	○	○	○	○	○
Skin Lesion/Rash	○	○	○	○	○	○
Acute care (URI/UTI/etc.)	○	○	○	○	○	○
Hospital/ED follow-up	○	○	○	○	○	○
COVID-19 Assessment	○	○	○	○	○	○

3. Telehealth visits add value to my practice.

Strongly Disagree	Disagree	Neutral	Agree	Strongly Agree	N/A
○	○	○	○	○	○

4. A telehealth visit is more efficient than an in-person visit.

Strongly Disagree	Disagree	Neutral	Agree	Strongly Agree	N/A
○	○	○	○	○	○

5. Being able to do a limited physical exam during a video visit adds value over a telephone visit alone.

Strongly Disagree	Disagree	Neutral	Agree	Strongly Agree	N/A
○	○	○	○	○	○

6. What are your biggest challenges, if any, that you face regarding telehealth visits? Select all that apply.

- [ ] Lack of reimbursement
- [ ] Licensure
- [ ] Technology challenges for the patient
- [ ] Low patient engagement
- [ ] Lack of implementation support
- [ ] Technology challenges for the provider/practice
- [ ] No challenges
- [ ] Other (please specify)

7. Telehealth has impacted my practice by improving ______________.

	Strongly Disagree	Disagree	Neutral	Agree	Strongly Agree	N/A
the health of my patients	○	○	○	○	○	○
the safety of my patients	○	○	○	○	○	○
timeliness of care of my patients	○	○	○	○	○	○
patient and family centered care	○	○	○	○	○	○
access to care	○	○	○	○	○	○
health of my patients	○	○	○	○	○	○
no show rate	○	○	○	○	○	○
financial health of my practice	○	○	○	○	○	○
satisfaction of my job	○	○	○	○	○	○

8. In the past 3 months, have you experienced any technical issues when using telehealth technology?

○ Yes

○ No

MUNSON HEALTHCARE

Telehealth Research Project

9. What issues have you encountered with telehealth visits? Check all that apply:

- ☐ Video not working at all
- ☐ Video stopped working in the middle of the visit
- ☐ Video working but inadequate for what I needed to look at
- ☐ Audio stopped working in the middle of the visit
- ☐ Audio working but inadequate for what I needed to hear
- ☐ Patient had problems connecting to the service
- ☐ I had problems connecting to the service
- ☐ Telehealth technology failed
- ☐ Other (please specify)

10. What did you do to remediate? Check all that apply:

- ☐ Resorted to a telephone call
- ☐ Asked the patient to reschedule for video visit
- ☐ Used another video visit technology
- ☐ Asked the patient to reschedule for in-person visit
- ☐ Other (please specify)

MUNSON HEALTHCARE

Telehealth Research Project

11. Moving forward, what percentage of your visits do you think will be virtual?

- ◯ 0
- ◯ 1-10%
- ◯ 11-20%
- ◯ 21-40%
- ◯ 41-60%
- ◯ >60

12. Do you practice at a Munson Healthcare owned practice?

- ◯ Yes
- ◯ No

13. Age Range of Provider

- ◯ 25-30
- ◯ 31-40
- ◯ 41-50
- ◯ 51-65
- ◯ >65

14. How long have you been practicing medicine?

- ◯ Resident
- ◯ <5 years
- ◯ 5-10 years
- ◯ 11-20 years
- ◯ 21-30 years
- ◯ >30 years

MUNSON HEALTHCARE

Telehealth Research Project

Thank you for taking the time to complete this survey.
